# Influence of Ionization and the Addition of Cyclodextrins and Hydrophilic Excipients on the Solubility of Benzthiazide, Isoxicam, and Piroxicam

**DOI:** 10.3390/pharmaceutics17050571

**Published:** 2025-04-25

**Authors:** Diego Lucero-Borja, Rebeca Ruiz, Elisabet Fuguet, Clara Ràfols

**Affiliations:** 1 Departament d’Enginyeria Química i Química Analítica and Institut of Biomedicina (IBUB), Universitat de Barcelona, Martí i Franquès 1-11, 08028 Barcelona, Spain; c6h12o6n@gmail.com; 2 Pion Inc. (UK) Ltd., Forest Row Business Park, Forest Row, East Sussex RH18 5DW, UK; rruiz@pion-inc.com; 3 Serra Húnter Programme, Generalitat de Catalunya, 08002 Barcelona, Spain

**Keywords:** solubility, supersaturation, shake-flask, CheqSol, excipients, cyclodextrins, hydrophilic polymers, benzthiazide, isoxicam, piroxicam

## Abstract

**Background**: The bioavailability of a drug depends, among other parameters, on solubility. One of the strategies used to enhance the solubility of sparingly soluble drugs is the use of excipients. Excipients can interact with the drug by increasing its solubility and/or stabilizing supersaturated solutions. Some of the most common excipients are cyclodextrins and hydrophilic polymers. **Objectives**: The effect of two cyclodextrins (captisol and cavasol) and three hydrophilic polymers (klucel, kollidon and plasdone S630) on the solubility of three ionizable drugs (benzthiazide, isoxicam, and piroxicam) is evaluated at biorelevant pH values, using two complementary techniques. **Methods**: The solubility enhancement was evaluated by the comparison of the solubility with and without the presence of excipients through the shake-flask and CheqSol methodology. **Results**: Captisol and cavasol slightly increase the concentration of the neutral species of the drugs in the solution before precipitation begins, although they do not enhance the supersaturation duration nor the thermodynamic solubility of the drugs. The increase in solubility in the presence of cyclodextrins is mainly caused by the ionization state of the drug. Hydrophilic polymers not only improve thermodynamic solubility but also the extent and the duration of the supersaturation. Some metastable forms are observed for benzthiazide and isoxicam in the presence of kollidon and plasdone S630. **Conclusions**: The shake-flask method enabled the evaluation of thermodynamic solubility both in the absence and presence of excipients. Meanwhile, the CheqSol method provided insights into the presence of supersaturated solutions. Different behavior is observed depending on the nature of the excipient.

## 1. Introduction

The importance of solubility as a physicochemical parameter in the bioavailability of drugs is well known [1,2]. Only a drug in a solution can be absorbed through the gastrointestinal tract (GIT); nevertheless, a high number of drug candidates is poorly soluble not only in water itself but also in the presence of gastrointestinal medium components. The solubility of ionizable drugs is pH-dependent, with the ionic form being more soluble than the neutral one. Then, the solubility of a drug can change through the GIT depending on the pH of the GIT zone and the pK_a_ of the drug [3,4,5,6]. Several strategies can be employed to improve the solubility of drugs and consequently their bioavailability. Among the strategies used for compounds classified in group II on the Biopharmaceutics Classification System (BCS) [7,8], which present solubility-limited absorption, the creation of a supersaturation solution through the incorporation of solubilizing agents is the most popular. Compounds that can supersaturate have low aqueous thermodynamic solubility and a strong ability to supersaturate, making them ideal candidates for assessing the potential impact of supersaturation effects on bioavailability in the gastrointestinal tract, as noted by Kostewicz et al. [9]. The key is to maintain the drug in its supersaturated state for long enough to afford the drug time to be absorbed in the system. Solubilizing agents enhance the drug solubility mainly by interaction with the drug [10,11,12,13,14]. The use of appropriate excipients in the drug formulation can act as stabilizers of supersaturated solutions and/or as enhancers of solubility. Hydrophilic polymers tend to keep the drug in the supersaturated phase by the inhibition of the precipitation process. In contrast, cyclodextrins, which form an inclusion complex, are mainly used as solubilize agents [13,14,15,16,17]. Many excipients are used in the pharmaceutical industry. For instance, hydrophilic polymers, like hydroxypropylcellulose (HPC) and polyvinylpyrrolidones (PVP), are widely used due to their ability to increase the dispersibility of and hydrogen bond formation with the drug. HPC is a cellulose polymer derivative, and some commercial variations are available depending on the desired size of the polymer. The chemical structure of PVPs consists of a pyrrolidone ring linked to a vinyl group, which acts as a bridge to the subsequent unit. A polymer is formed by the repetition of “N” such units, and the polymer exhibits a different hydrophilic degree by placing different radical groups or vinyl groups in the ring. The physicochemical properties of PVPs can be tuned by copolymerization with other polymeric subunits. This is the case, for example, of plasdone S630, which presents a random combination of vinylpyrrolidone and vinylacetate units. Cyclodextrins (CDs) are structures based on modified glucoside groups adopting a toroidal formation that creates a hydrophobic internal cavity while maintaining a sufficiently hydrophilic external surface. The cavity size varies depending on the specific type of CD. β-CDs are the ones commonly utilized in pharmaceutical applications, and their inner cavity can be tailored through the modification of functional groups [13,18,19,20,21,22,23,24]. While all of these excipients influence drug solubility, predicting the behavior of a particular excipient in a solution remains challenging due to the numerous factors affecting the excipient–drug complex, including the formulation design, absorption site, drug solubility, and stability. The knowledge of these type of interactions can help to understand the enhancement in solubility and their effect in drug absorption [1,13].

The effect of the excipients on the solubility of the drug can be evaluated by the comparison of the solubility of the drug with and without the presence of excipients using two complementary methods: shake-flask and CheqSol [25,26,27]. Shake-flask is the traditional method to reach the thermodynamic equilibrium between the solid and the aqueous form of a certain compound. Working with an appropriate buffer, the solubility of the drug is obtained at fixed pH value. Moreover, this method easily allows the solid collection after the separation of the two phases for its identification. The CheqSol method can only be applied for ionizable compounds. The method potentiometrically measures the solubility of the neutral species (intrinsic solubility, S_0_). Moreover, it provides information about the ability of the drug to form supersaturated solutions, indicating the degree of the supersaturation and the time range in which the solution is supersaturated.

In this study, five excipients were chosen to evaluate their impact on the solubility of three pharmaceutical compounds (see Figure S1). The selected excipients included two cyclodextrins (Captisol and Cavasol) and three polymers (Klucel, Kollidon, and Plasdone S630). The selected drugs were benzthiazide, isoxicam, and piroxicam. Benzthiazide is a thiazide diuretic used in the treatment of high blood pressure and edema. It has two acidic ionizable groups, one in a benzothiadiazine ring and another in the sulfamide group. Isoxicam and piroxicam are nonsteroidal anti-inflammatory drugs (NSAIDs) of the oxicam class used to reduce inflammation. Isoxicam is a monoprotic acid and piroxicam an ampholyte. Both have a monocarboxylic acid amide (acidic group), with isoxicam being a chemical analog of piroxicam in which a pyridine ring is replaced by an isoxazole ring. Moreover, piroxicam has a basic ionizable group in the nitrogen of the pyridine ring. Table 1 shows the chemical structure and some physicochemical properties of the selected compound.

The main purpose of this work is, therefore, to evaluate the effect of a set of excipients of different nature and that are commonly used in drug formulation studies on the solubility of the three drugs at three biorelevant pH values. This evaluation was conducted from two different perspectives: In the first instance, the increase in the thermodynamic solubility of the drugs was evaluated using the shake-flask method. In a second study based on the CheqSol method, the ability of the excipients to stabilize supersaturated solutions was also checked.

## 2. Materials and Methods

### 2.1. Reagents

The APIs used, benzthiazide (>99%), isoxicam (>99%), and piroxicam (>98%), were from Sigma Aldrich (Darmstadt, Germany). The excipients, all of them of pharmaceutical grade, were: Captisol^®^ (β-cyclodextrin sulfobutyl ether, sodium salt, MW ~ 2163 g mol^−1^, degree of substitution 6.5) (CAP) provided by Ligand Pharmaceuticals (San Diego, CA, USA); cavasol ((2-hydroxypropyl)-β-cyclodextrin, MW ~ 1410 g mol^−1^, degree of substitution 4.1–5.1) (CAV) and klucel (hydroxypropyl cellulose, MW ~ 80,000 g mol^−1^) (KLU) from Sigma-Aldrich; kollidon^®^ 17PF (MW ~ 9000 g mol^−1^) (KOL) from BASF (Ludwigshafen, Germany) and provided by BTC_CI (Burgbernheim, Germany); and PVP S630 (MW ~ 43,000 g mol^−1^), a 60:40 random copolymer of vinylpyrrolidone and vinylacetate, from International Specialty Products (Wayne, NJ, USA), and provided by Ashland (Covington, KY, USA).

Maleic acid > 99.5% (Carlo Erba, Milan, Italy), sodium chloride (Fisher Scientific, Loughborough, UK), potassium hydroxide 1 M Titrisol^®^, and hydrochloric acid 1 M Titrisol^®^ from Merck (Darmstadt, Germany) were used for the preparation of buffers in the shake-flask measurements.

Methanol (HPLC gradient-grade) from Chem-Lab NV (Zendelgem, Belgium) and sodium dihydrogen phosphate monohydrate (>99%) and disodium monohydrogen phosphate (>99%) from Merck were used for HPLC quantification.

Dimethyl sulfoxide (≥99.5%) and methanol (≥99.8%) (from VWR (Leicestershire, UK)), and 0.5 M potassium hydroxide (titrisol), 0.5 M hydrochloric acid (Titripur), potassium chloride (≥99%), potassium hydrogen phthalate (>99%), and potassium phosphate dibasic trihydrate (≥99.0,), purchased from Sigma-Aldrich, were used in the CheqSol, pK_a_, and logP_o/w_ measurements. Additionally, n-octanol (HPLC grade, ≥99.9%,) from Sigma-Aldrich was also used for the logP_o/w_ determination.

Water was purified by the Milli-Q plus system from Millipore (Bedford, MA, USA), with a resistivity of 18.2 MΩ cm.

### 2.2. Instruments

#### 2.2.1. Shake-Flask Method

A GLP22 pH meter from Crison (Alella, Spain), equipped with an Ag/AgCl KFP-1087 Pion electrode, was utilized for the pH measurements of buffer solutions and samples. Sample agitation was carried out using P-SELECTA rotatory shakers, model MOVIL-ROD (Abrera, Spain). For the centrifugation process, a Rotanta 460RS centrifuge with temperature control (Hettich Lab Technology, Tuttlingen, Germany) was employed, operating at a fixed speed of 3500 rpm and a temperature of 25 °C.

A Shimadzu (Kyoto, Japan) liquid chromatograph, equipped with two Shimadzu LC-10ADvp pumps, a SIL-10ADvp autosampler, and a Shimadzu SPD-10AV detector was used for HPLC measurements, using a C18 Gemini column 150 × 4.6 mm and 5 μm particle size from Phenomenex (Alcobendas, Spain). The temperature was controlled at 25.0 ± 0.1 °C with a Shimadzu CTO-10AS column oven.

Powder X-ray diffraction (PXRD) measurements were performed with a A PANalytical X’Pert PRO MPD θ/θ diffractometer (Malvern, UK), with a 240 mm radius in the transmission configuration. The instrument utilized Cu Kα1 + 2 radiation (λ = 1.5406 Å) with a focusing elliptic mirror and a PIXcel detector. It was set to a maximum active length of 3.347° for the detector, employing a convergent beam with a focalizing mirror and transmission geometry, where the flat sample was sandwiched between low-absorbing films. The measurements were conducted across a 2θ range from 1° to 40°, with a step size of 0.026° and acquisition times of either 75 or 300 s per step.

#### 2.2.2. CheqSol Method

A SiriusT3 titrator system, from Pion Inc. (Billerica, MA, USA)., was used for the ionization constant (pK_a_) and potentiometric solubility (CheqSol) measurements. This system was equipped with an Ag/AgCl double junction reference pH electrode, a temperature controller, an overhead stirrer, and motorized dispensers for the automated delivery of assay titrants and reagents via capillaries. Additionally, the SiriusT3 incorporates a UV–Vis spectrophotometer equipped with a photodiode array detector, a deuterium lamp, and a fiber optic mini-dip probe for comprehensive spectrophotometric data collection.

The titrant standardization process involved the standardization of a 0.5 M potassium hydroxide base titrant using potassium hydrogen phthalate. Subsequently, a 0.5 M hydrochloric acid titrant was standardized against the base titrant. The pH electrode was calibrated daily in accordance with the Avdeef–Bucher four-parameter equation [29].

The instrumentation was controlled via the SiriusT3Control software (V2.0), while data processing and the derivation of the reported pK_a_ and solubility values were performed using the SiriusT3Refine software (V2.0).

### 2.3. Procedures

#### 2.3.1. pK_a_ and logP_o/w_ Determination

The pK_a_ and logP_o/w_ determinations were performed using the SiriusT3 titrator (Pion Inc.). The titrations were carried out from pH 12.0 to 2.0 due to the acidic nature of the drugs, at an ionic strength of 0.15 M (KCl), under an argon atmosphere and controlled temperature (25.0 ± 0.1 °C).

The pK_a_ was determined with the spectrometric UV-metric method, at concentrations between 32 µM and 23 µM in aqueous media. For the analysis, 5 μL of a 10 mM DMSO sample stock solution, and 25 μL of a 15 mM potassium phosphate buffer, were added to 1.5 mL of a 0.15 M KCl solution. The UV-metric titration method used the UV absorbance changes in the aromatic groups close to the ionizable groups of the substance as a function of pH [30]. The compounds studied contained acidic ionizable groups; hence the substances were dissolved in their ionized form at a high pH and subsequently back titrated to create the free acid form of the drug. The pK_a_s were determined from the absorbance of the species, acquired between 240 and 400 nm, at different pH values through target factor analysis. Results are the mean value from four triple titrations.

Octanol–water partition values, logP_o/w_, were obtained by potentiometric titrations in the presence of octanol at concentrations between 1.4 mM and 0.4 mM. The logP_o/w_ was calculated by the difference between the aqueous pK_a_ and the apparent p_o_K_a_ (pK_a_ measured in the presence of octanol) at several phase ratios (octanol:water) between 0.04:1 and 1.70:1. At least five titrations were made for each compound [31,32].

#### 2.3.2. Shake-Flask Solubility Determination

Prior to performing the solubility measurements, maleic/maleate buffers were prepared as follows: a stock solution containing 55 mM maleic acid, 126 mM sodium chloride, and 82 mM NaOH (conditions that mimic the fed state of the intestinal fluid) was made following the guidelines of Biorelevant [33]. Final buffers at pH 2, 5.8, and 6.5 were prepared by the addition of HCl or NaOH to three different aliquots of the stock solution.

The solubilities of benzthiazide, isoxicam, and piroxicam and their corresponding mixtures with excipients were determined by the shake-flask method, following the consensus recommendations described previously by Avdeef et al. [26]. The mixtures were prepared at a 1:1 weight ratio of excipient:API by grinding gently in a mortar until obtaining a homogeneous mixture. Enough amount of the sample (between 5 and 15 mg) was weighed in a glass tube of 5 mL, and 3 mL of buffer solution were added. The weighed amount must be enough to obtain saturated solutions, which depends on the sample and pH of the buffer used. The samples were shaken for 24 h at 25 ± 0.5 °C, with a pH control of the solutions after 4 h of shaking. If necessary, the pH was readjusted to the desired value with small volumes of 1 M HCl or 1 M NaOH. After shaking for 24 h, the samples were allowed to equilibrate for an additional 24 h. Subsequently, the final pH was measured, and phase separation was carried out via centrifugation. The supernatant was collected for drug quantification, while the remaining solid was dried under vacuum for 30 min and stored at 4 °C until PXRD analysis. In all cases, the solid-state analysis confirmed that there were no transformations, as the final solid form of the API was the same as the commercial initial form (Figure S2 of the Supplementary Information shows, as an example, the results obtained for benzthiazide at pH 5.8).

The quantification of the supernatant was performed by liquid chromatography under isocratic conditions using methanol/10 mM phosphate buffer at pH 7.5 as the mobile phase. The compositions for piroxicam and isoxicam quantification were 50/50 (*v*/*v*), whereas for that for benzthiazide was 45/55 (*v*/*v*). The flow rate was set at 1 mL/min, with an injection volume of 10 μL. Detection wavelengths were set at 319 nm for benzthiazide, 350 nm for piroxicam, and 357 nm for isoxicam. Five standard solutions were prepared in the linear range of each compound (0.5–50 mgL^−1^, 0.2–8 mgL^−1^, and 5–250 mgL^−1^ for benzthiazide, piroxicam, and isoxicam, respectively). If necessary, an appropriate dilution in water of the saturated supernatant was carried out to ensure its fit in the range of calibration.

#### 2.3.3. CheqSol Solubility Determination

For the determination of the intrinsic solubility using the potentiometric method [27], two events must happen: First, the compound must be fully dissolved at the beginning of the titration. Second, the precipitation of the compound from the solution should occur close to the equivalent point of the titration curve. The amount of compound used in a CheqSol determination must be enough to achieve both events. Hence, depending on the compound solubility, the nature of the excipient, and the interaction of drug–excipient, the amount of drug must be adjusted. Taking into account that the ratio of drug:excipient selected for this study was 1:1, the starting concentration of the assay was between 8.5 mM and 3.5 mM for benzthiazide, 18.9–5.3 mM for isoxicam, and 15.3–7.0 mM for piroxicam. All titrations started at pH 12.0, where the compounds were ionized and titrated to a low pH to force precipitation. In this study, the drugs precipitated from solutions in the range of pH 6.6–8.7 for benzthiazide, pH 3.7–6.7 for isoxicam, and pH 5.7–6.7 for piroxicam. The precipitation was detected when the absorbance readings were higher than 0.1 absorbance units at 500 nm by a UV–turbidity probe, and after precipitation, base and acid titrants were alternately added to drive the sample back and forth across the equilibrium solubility of the neutral species (the intrinsic solubility). At this point, the sample existed in a supersaturated or subsaturated state (i.e., chase equilibrium). The change between the subsaturated and supersaturated states was repeated several times to determine the intrinsic solubility by calculating the pH by interpolation between the two saturated states. The intrinsic solubility was calculated using the principles of mass and charge balance at the pH at which the equilibrium of the system was reached [27]. All solubilities were determined at least in quadruplicate at the average ionic strength of 0.17 M (KCl), under an argon atmosphere and controlled temperature (25.0 ± 0.1 °C).

## 3. Results and Discussion

The influence of five excipients on the solubility of three acidic compounds—benzthiazide, isoxicam, and piroxicam—was evaluated at physiologically relevant pH levels using both the shake-flask and potentiometric CheqSol methods. The selected excipients consisted of two cyclodextrins (captisol and cavasol) and three hydrophilic polymers (klucel, kollidon, and plasdone S630). The pH values examined were 2.0, 5.8, and 6.5. The first one was used to simulate the pH of the stomach, whereas pH 5.8 and 6.5 simulated the pH values at different duodenal stages, the fed and fasted states, respectively.

According to compounds’ pK_a_ values (Table 1), the measurement of the solubility at pH 2 provided the intrinsic solubility (S_0_) of benzthiazide and isoxicam, whereas for piroxicam, S_0_ was determined at pH 3.5, where the zwitterionic form of the compound predominated over the other forms. Therefore, this pH value was also included in the study.

### 3.1. Shake-Flask Determinations

**Figure 1:**
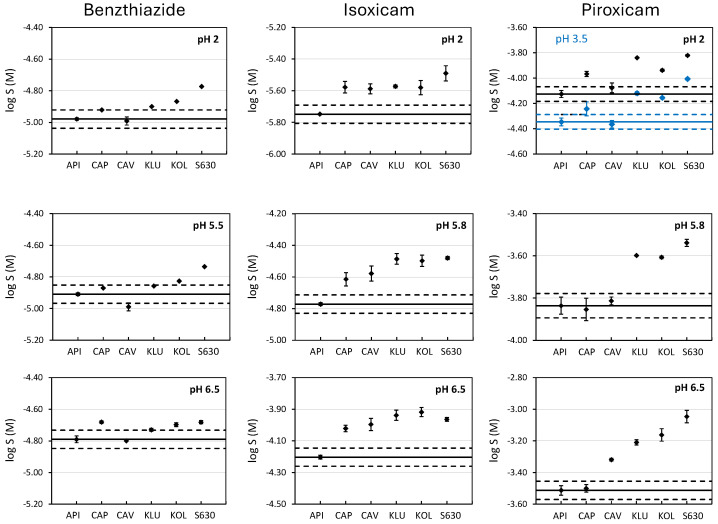
Solubility of benzthiazide, isoxicam, and piroxicam at pH 2, 5.8, and 6.5 without and with the presence of excipients. Black dots correspond to the experimental solubility at pH, 2, 5.8, and 6.5. For piroxicam, the blue dots correspond to the solubility at pH 3.5. The straight line represents the solubility of the compound without excipient (active pharmaceutical ingredient, API) and the dashed lines indicate the range log S_0_ ± 0.1. Excipients: Captisol (CAP), Cavasol (CAV), Klucel (KLU), Kollidon (KOL), and PVP S630 (S630).

Figure 1 and Table S1 show the solubility values of benzthiazide, isoxicam, and piroxicam determined at the three studied pHs without and with the presence of an excipient (formulated at 50% *w*/*w* relative to the API). When the solubility of the free drugs (without excipients) is evaluated, it can be observed that the intrinsic solubility is in agreement with their hydrophobicity (Table 1). Piroxicam, which has the lowest logP_o/w_ value, is the most soluble, while isoxicam, being the most hydrophobic, is the least soluble. Benzthiazide has an intermediate solubility. Moreover, as expected, the solubility of the compounds increases with the ionization degree. For instance, the solubility of benzthiazide practically does not change with the pH until the ionization of the compound is around 40% (pH 6.5). Here, a slight increase is observed. Isoxicam is fully ionized at pH 5.8; hence, the solubility observed at this pH is higher than its solubility at pH 2.0. The pK_a1_ of piroxicam is attributed to the nitrogen atom located in the pyridine ring. Therefore, at pH 2, this molecule is partially ionized in its cationic form (around 40%), and its solubility is higher than that at pH 3.5, where the molecule is practically in its neutral form. At pH 5.8 and 6.5, the solubility increases due to the presence of the anionic form of the drug.

The effect of a given excipient on the solubility of a drug depends on both the nature of the compound and the nature of the excipient used. To evaluate if the enhancement in solubility is significant with respect to the solubility in water when the excipients are added, the criterion of twice the standard deviation (sd) was applied. That is, an enhancement is considered when the solubility value in the presence of an excipient is higher than logS_API_ ± 2 sd, where S_API_ is the solubility of the drug in aqueous buffers (without excipient) and a sd value of 0.05 is used for all the pHs studied. This value was chosen because it corresponds to an average standard deviation obtained in our laboratory for shake-flask solubility determinations and it also agrees with typical sd reported in the literature [26,34]. Table 2 shows the enhancement in the solubility caused by the presence of excipients (S_EXC_) expressed as the relation between both solubilities (S_EXC_/S_API_).

The enhancement in the solubility of neutral compounds due to the presence of cyclodextrins (CAP and CAV) is mainly attributed to the hydrophobic interactions driven by the inclusion of the molecules inside of its cavity. The larger the cavity, the better the inclusion of the drug [18,19,20]. Whereas the sulfonic groups of CAP are repulsing each other enlarging the entrance of the cavity, for CAV, the hydroxyl groups are closer, making the size of the cavity smaller (Figure S1). This effect can explain the different behavior observed on the solubility of benzthiazide, which is the compound with the highest molar volume (Figure 1 and Table 2), as no solubility enhancement is observed in the presence of CAV; instead, a small enhancement is observed in the presence of CAP, independently of the pH. This indicates that the interactions may happen through the non-substituted benzene ring, with higher hydrophobicity than the other side of the molecule where the two ionizable groups are located. In the case of isoxicam, a similar and significant enhancement (around 1.5, Table 2) is observed in the presence of both CAP and CAV, whatever the tested pH. In fact, this molecule has a lower molar volume compared to benzthiazide, and this facilitates the interaction with CAV. A similar solubility enhancement is observed independently of the ionization degree (isoxicam is neutral at pH 2, and practically 100% ionized at pH 5.8 and 6.5). This suggests that interactions happen through the formation of inclusion complexes driven by hydrophobic interactions (notice that it is the compound with the highest logP_o/w_ value, Table 1) by the side of the molecule opposite to the ionizable group. However, this hypothesis is not supported by the piroxicam data. Piroxicam, with practically the same molar volume as that of isoxicam, hardly interacts with CAV. This behavior can only be explained if the interaction is not due to the formation of inclusion complexes, but through external hydrogen bonding interactions. In fact, Goswami et al. [35] demonstrated the presence of hydrogen bond interactions between the isoxazole ring from isoxicam and the external hydroxyl groups of CAV. Because the pyridinic ring of piroxicam has only one hydrogen bond acceptor heteroatom, these interactions can be lower for isoxicam. The solubility enhancement in piroxicam in the presence of CAP is strongly marked by the ionization degree of the API. At pH 2, the compound is almost 40% positively charged and the highest solubility enhancement is observed, possibly due to electrostatic interactions between the positive charges and the sulfonic groups of CAP. At pH 3.5, where the API is mostly neutral, only a slight enhancement is observed, most likely due to hydrophobic interactions. Finally, the solubility is not increased at pH 5.8 nor 6.5, where piroxicam is negatively charged (75% and 96%, respectively).

Hydrophilic polymeric excipients show a different behavior compared to cyclodextrins. Klucel, a hydroxypropylcellulose with numerous hydroxyl groups, is a hydrogen bond (HB) donor, whereas kollidon and plasdone S630, both with polyvinylpyrrolidone groups, have HB acceptor capability. The main difference between kollidon and plasdone S630 is the presence of vinylacetate groups in the plasdone, which increase the HB acceptor capacity of this excipient (Figure S1). Because the three studied molecules have HB acceptor and donor groups (see Table 1), they should be able to interact with the three excipients, which would improve the solubility. This can be observed in Figure 1 and Table 2. The three compounds experience a moderate increase in solubility (up to 3-fold increase), especially in the presence of the two polyvinylpyrrolidones (Kollidon and plasdone S630). The improvement is slightly better in the presence of plasdone S630, which has a higher HB acceptor ability. Whereas the pH is an important factor in the interactions of the drugs with cyclodextrins, with polymeric excipients, the effect of the pH is somehow lowered, as the solubility is enhanced due to drug–excipients interactions regardless of the ionization degree of the drugs.

### 3.2. CheqSol Determinations

**Figure 2:**
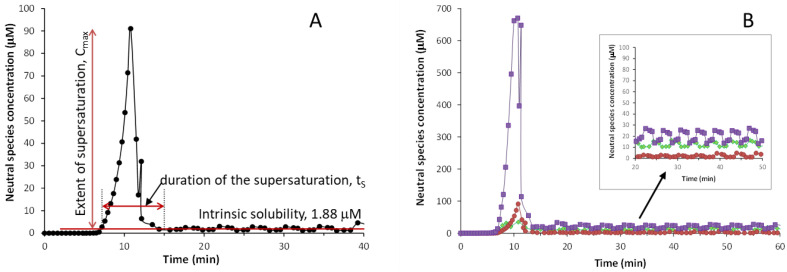
Concentration of the neutral species vs time plots for: (**A**) isoxicam, (**B**) benzthiazide (♦), isoxicam (●), and piroxicam (■).

The CheqSol method not only provides the thermodynamic solubility of the neutral form (S_0_) of an ionizable compound but also the extent and time that a solution remains supersaturated before reaching S_0_. Both parameters are used to evaluate the supersaturation of a compound [10]. In this method, the experiment starts in conditions where the drug is completely ionized, and so, fully dissolved. Therefore, the concentration of the neutral species in solution is “zero”. The titration begins by titrating towards the pK_a_ of the drug, to force precipitation as the neutral species starts to form. During this process, the concentration of the neutral species in solution increases to a maximum (C_max_) before the drug precipitates. When precipitation starts, it decreases dramatically to a much lower concentration to reach the intrinsic solubility. It must be noted that, before the drug precipitates, the solution is supersaturated. However, this supersaturated solution is thermodynamically unstable and has the tendency to quickly return to the equilibrium state by precipitation to the most stable form of the drug. The supersaturation parameters can be obtained from the plot that monitors the concentration of neutral species in solution against time. Figure 2A shows the plot obtained for isoxicam, which displays the concentration in solution of the neutral species of isoxicam collected during the CheqSol process. The extent of the supersaturation corresponds to the maximum concentration of the neutral species in solution (C_max_) before the drug precipitates, and the duration of the supersaturation (t_s_) is the amount of time that the drug stays supersaturated until it reaches equilibrium solubility. In addition, the supersaturation ratio (R_s_) can be calculated by dividing C_max_ by the intrinsic solubility (S_0_).

Figure 2B compares the supersaturation behavior of the three studied compounds. The S_0_ values determined for the three compounds by CheqSol follow the same trend as the ones obtained by the shake-flask method, i.e., isoxicam has the lowest S_0_ value (logS_0_ = −5.72 (0.01)), followed by benzthiazide (log S_0_ = −4.95 (0.03)), and piroxicam is the one with the highest solubility (log S_0_ = −4.67 (0.03)). The values obtained are in agreement with the ones obtained by the shake-flask method (Table S1). Regarding supersaturation, the oxicam compounds present a higher supersaturation than benzthiazide, with the C_max_ of piroxicam being much higher than that of isoxicam. Nevertheless, the duration of the supersaturation is short for the three compounds, around 10 min.

**Figure 3:**
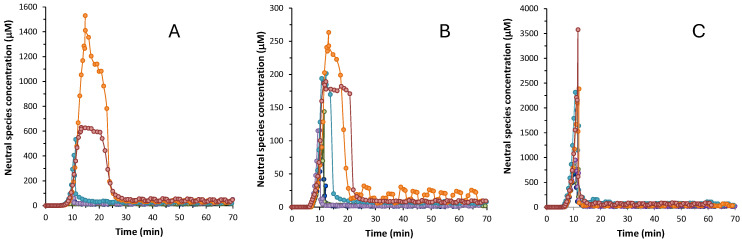
Concentration of the neutral species vs time plots for (**A**) benzthiazide; (**B**) isoxicam; (**C**) piroxicam. (●) Without excipient, (●) with captisol, (●) with cavasol, (●) with klucel, (●) with kollidon, and (●) with plasdone S630.

The influence of the excipients on the supersaturation is shown in Figure 3 and Figure S3. Table 3 reports the supersaturation parameters of the three drugs in the presence and absence of excipients: C_max_, t_s_, and R_s_. In order to visualize the overall increase in solubility during supersaturation, R_s_ was calculated by the quotient between C_max_ and S_0_ of the free drug, in the absence of any excipient. All three drugs exhibit a certain degree of supersaturation before the precipitation of the most stable form. The highest supersaturation is observed for isoxicam that, in absence of any excipient, can stay supersaturated at a concentration 48-fold higher than its intrinsic solubility for a reduced period of time (11 min). All three drugs present enhanced supersaturation profiles in the presence of excipients. However, these profiles are markedly different depending on whether cyclodextrins or hydrophilic excipients are used. The use of cyclodextrins does not result in significant differences in the supersaturation behavior of none of the three drugs, compared to the profile without excipient (Figure S3): the C_max_ increases only slightly (around 1.5 times) compared to C_max_ without the excipient. Moreover, the duration of the supersaturation is practically the same as for the free APIs. (around 7–8 min). Instead, the use of hydrophilic excipients, where the drug is dispersed within the polymer structure, has more influence in the supersaturation process. They provide a higher C_max_ and higher supersaturation ratio for the three compounds. The highest C_max_ values for benzthiazide and isoxicam are achieved with kollidon (1404 and 317 μM, respectively), whereas for piroxicam, C_max_ is obtained in the presence of plasdone S630 (3561 μM). The supersaturation ratio R_S_ is significantly higher with these polymeric excipients compared to cyclodextrins. For instance, the Rs of benzthiazide is 40-fold higher in the presence of klucel and plasdone S630, and 100-fold higher in the presence of kollidon. The increase is not so marked for isoxicam and piroxicam, but the supersaturated solutions are between 2-fold and 5-fold higher in the presence of these excipients. This means that polymeric excipients enable a higher concentration of the drug in solution before precipitation begins. The hydrophilic excipients not only provide a higher C_max_ and supersaturation ratio, but also a higher time of supersaturation (~20 min for benzthiazide and isoxicam and ~10 min for piroxicam). Among the three hydrophilic excipients tested, polyvinylpyrrolidones (kollidon and plasdone S630) show a distinctive behavior with benzthiazide and isoxicam, as they increase the extent and duration of the supersaturation (Figure S3A,B; Table 3). It is quite likely that these two compounds have a metastable form stabilized by the interactions with these excipients. These metastable forms remain in solution for around ~20 min before the most stable form finally precipitates. This behavior was already observed with other polyvinylpirrolidones, which stabilized metastable forms of acidic compounds [10]. Isoxicam also shows an initial metastable form in the presence of klucel (Figure 3B and Figure S3B), but it stabilized only for a short period of time (around 16 min). The formation of metastable forms is also observed in the case of benzthiazide (Figure 3A and Figure S3A). On the contrary, piroxicam has an increase in the supersaturation ratio, but does not show a metastable form (Figure 3C and Figure S3C). Similar to cyclodextrins, in the presence of hydrophilic excipients, piroxicam precipitates rapidly in the most stable form to reach equilibrium.

## 4. Conclusions

The combined use of the shake-flask and the CheqSol methods have allowed the evaluation of the effect of two cyclodextrins (cavasol and captisol) and three hydrophilic polymers (klucel, kollidon and plasdone S630) on the solubility of benzthiazide, isoxicam, and piroxicam, in terms of thermodynamic solubility and capacity to form supersaturated solutions. In their neutral state and in the absence of excipients, supersaturated solutions of the neutral form are only maintained for a limited period of time. All three compounds have ionizable groups; so, their solubility is pH-dependent, increasing with the degree of ionization.

The use of cyclodextrins has practically no effect on the solubility of benzthiazide and piroxicam. Specifically, cavasol does not enhance their solubility because they have a molar volume that prevents them from entering the cavity. Captisol, with a larger cavity, slightly improves the solubility of both drugs. In the case of isoxicam, with a molar volume similar to that of piroxicam, an increase in solubility is observed with both cavasol and captisol, likely attributable to hydrogen bond interactions (HBs) on the surface of the cyclodextrin. The interactions with cyclodextrins do not change with the ionization degree, except in the case of piroxicam when it is in its cationic form. Under these conditions, an increase in its solubility is observed in the presence of captisol, attributable to electrostatic interactions between the positive charge of the drug and the negative charge of the cyclodextrin. In terms of supersaturation behavior, a slight increase in the concentration is observed (C_max_) before the drug starts precipitation, although cyclodextrins do not allow the stabilization of supersaturation solutions.

The three drugs behave differently in the presence of polymeric excipients (klucel, kollidon, and plasdone S630) due to the ability of these excipients to interact through hydrogen bonds. As the three drugs have HB acceptor and donor groups, their solubility increases in the presence of these excipients, with the greatest increase in the presence of plasdone S630, which has the highest HB ability. Regarding supersaturation, the presence of polymers not only provides a higher C_max_ than that of cyclodextrins but also a higher supersaturation time. Nevertheless, a different behavior was observed depending on the polymer used and the drug studied. In general, it was observed that klucel, with the lowest capacity for HB formation, is the polymer that maintains the supersaturated solution for the shortest time, while kollidon and plasdone temporarily stabilize a metastable form of benzthiazide and isoxicam.

## Figures and Tables

**Table 1:** Physicochemical properties of the selected compounds at I = 0.15 M and 25 °C. Standard deviations in parenthesis.

Compound	Chemical Structure	pK_a_	log P_o/w_	Molar Volume ^2^ (cm^3^)
Benzthiazide	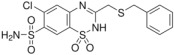	6.64 ^1^; 9.22 ^1^	1.89 (0.02)	259.0
Isoxicam	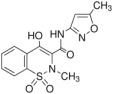	3.84 ^1^	2.99 (0.02)	211.0
Piroxicam	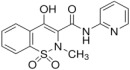	1.89 (0.01); 5.34 (0.01)	1.71 (0.02)	211.9

^1^ From ref. [10]; ^2^ from ref. [28].

**Table 2:** Enhancement in the solubility according to the pH and the excipient for the three studied APIs using the shake-flask methodology. 1:1 excipient:API weight ratio.

	Excipient	S_EXC_/S_API_		
		Benzthiazide	Isoxicam	Piroxicam
pH 2 ^1^	Captisol	1.14	1.48	1.44
	Cavasol	0.97	1.45	1.12
	Klucel	1.20	1.50	1.94
	Kollidon	1.29	1.47	1.54
	S630	1.61	1.81	2.02
pH 3.5 ^2^	Captisol	---	---	1.27
	Cavasol	---	---	0.96
	Klucel	---	---	1.69
	Kollidon	---	---	1.55
	S630	---	---	2.19
pH 5.8	Captisol	1.09	1.44	0.96
	Cavasol	0.83	1.56	1.25
	Klucel	1.12	1.93	1.73
	Kollidon	1.21	1.88	1.70
	S630	1.49	1.96	1.99
pH 6.5	Captisol	1.29	1.52	1.03
	Cavasol	0.98	1.61	1.56
	Klucel	1.15	1.84	2.01
	Kollidon	1.24	1.93	2.24
	S630	1.28	1.73	2.92

^1^ Neutral form for benzthiazide and isoxicam; ^2^ neutral form for piroxicam.

**Table 3:** Supersaturation parameters obtained through the CheqSol method: maximum measured concentration (C_max_), time of supersaturation (t_s_), and supersaturation ratio (R_s_). Standard deviation is shown in parenthesis.

Compound–Excipient	C_max_ (µM)	t_s_ (min)	R_s_
Benzthiazide	44 (11)	6 (1)	4 (1)
Benzthiazide–Captisol	50 (5)	6 (1)	4.2 (0.4)
Benzthiazide–Cavasol	92 (10)	7 (1)	7.7 (0.8)
Benzthiazide–Klucel	516 (54)	14 (2)	43 (5)
Benzthiazide–Kollidon	1404 (178)	21 (1)	104 (26)
Benzthiazide–Plasdone S630	535 (87)	25 (3)	45 (8)
Isoxicam	92 (9)	11 (3)	48 (4)
Isoxicam–Captisol	137 (6)	8 (1)	71 (3)
Isoxicam–Cavasol	122 (12)	7 (1)	64 (6)
Isoxicam–Klucel	195 (13)	16 (6)	102 (7)
Isoxicam–Kollidon	317 (73)	18 (4)	166 (38)
Isoxicam–Plasdone S630	200 (18)	19 (3)	105 (9)
Piroxicam	706 (135)	7 (1)	34 (6)
Piroxicam–Captisol	831 (56)	8 (1)	40 (3)
Piroxicam–Cavasol	849 (130)	7 (1)	41 (6)
Piroxicam–Klucel	2431 (454)	13 (2)	117 (22)
Piroxicam–Kollidon	1980 (450)	7 (1)	95 (22)
Piroxicam–Plasdone S630	3561 (576)	10 (1)	171 (28)

R_s_ = C_max_/S_0_, where S_0_ is the intrinsic solubility of the API in a medium without excipients.

## Data Availability

The raw data supporting the conclusions of this article will be made available by the authors upon request.

## Supplementary Materials

The following supporting information can be downloaded at: https://www.mdpi.com/article/10.3390/pharmaceutics17050571/s1, Table S1: Experimental log S values for benzthiazide, isoxicam, and piroxicam at different pH values in aqueous media, without and with addition of 50% (*w*/*w*) of excipient, determined using the shake-flask methodology; Figure S1: Structures of the excipients; Figure S2: PXRD diffractograms for benzthiazide raw material and for the solid collected after the shake-flask process at pH 5.8; Figure S3: Supersaturation profiles for benzthiazide, isoxicam, and piroxicam.

## Abbreviations

The following abbreviations are used in this manuscript:

API	Active pharmaceutical ingredient
EXC	Excipient
CAV	Cavasol
CAP	Captisol
KLU	Klucel
KOL	Kollidon
S630	Plasdone S630
