# Supplementary material for: Influence of Ionization and the Addition of Cyclodextrins and Hydrophilic Excipients on the Solubility of Benzthiazide, Isoxicam, and Piroxicam
Source: Pharmaceutics. 2025 Apr 25;17(5):571. doi: 10.3390/pharmaceutics17050571 (PMC12114812; doi:10.3390/pharmaceutics17050571)
Supplement: Supplementary file 1 [file pharmaceutics-17-00571-s001.zip › pharmaceutics-3566568-supplementary.pdf]

Influence of Ionization and the Addition of Cyclodextrins and Hydrophilic Excipients on the Solubility of Benzthiazide, Isox-icam, and Piroxicam

Diego Lucero-Borja 1, Rebeca Ruiz 2, Elisabet Fuguet 1,3,* and Clara Ràfols 1,*

[1] Departament d'Enginyeria Química i Química Analítica and Institut of Biomedicina (IBUB),

Universitat de Barcelona, Martí i Franquès 1-11, 08028 Barcelona, Spain

[2] Pion Inc. (UK) Ltd., Forest Row Business Park, Forest Row, East Sussex RH18 5DW, UK

[3] Serra Húnter Programme, Generalitat de Catalunya, 08002 Barcelona, Spain

* Correspondence: elifuguetj@ub.edu (E.F.); crafols@ub.edu (C.R.)

Table S1: Experimental log S values for benzthiazide, isoxicam and piroxicam at different pH values in aqueous media, without and with addition of 50% (w/w) of excipient, determined using the shake-flask methodology.

	Benzthiazide		Isoxicam		Piroxicam	
**Excipient**	**pH**	**log S (M)**	**pH**	**log S (M)**	**pH**	**log S (M)**
Without	2.14 (0.01)	-4.98 (0.01)	1.96 (0.01)	-5.75 (0.01)	2.00 (0.05)	-4.13 (0.03)
Captisol	2.10 (0.01)	-4.92 (0.01)	1.96 (0.06)	-5.58 (0.04)	1.99 (0.04)	-3.97 (0.02)
Cavasol	2.37 (0.01)	-4.99 (0.03)	1.96 (0.03)	-5.59 (0.03)	2.02 (0.06)	-4.08 (0.04)
Klucel	2.20 (0.01)	-4.90 (0.01)	1.96 (0.09)	-5.57 (0.01)	2.05 (0.01)	-3.84 (0.01)
Kollidon	2.20 (0.01)	-4.87 (0.01)	1.91 (0.01)	-5.58 (0.05)	2.05 (0.01)	-3.94 (0.01)
P-S630	1.96 (0.01)	-4.77 (0.01)	1.98 (0.01)	-5.49 (0.05)	2.04 (0.02)	-3.82 (0.01)
Without	5.55 (0.01)	-4.91 (0.01)	5.83 (0.02)	-4.77 (0.01)	5.85 (0.01)	-3.84 (0.04)
Captisol	5.55 (0.01)	-4.87 (0.02)	5.84 (0.03)	-4.61 (0.04)	5.86 (0.01)	-3.85 (0.05)
Cavasol	5.55 (0.01)	-4.99 (0.01)	5.85 (0.03)	-4.58 (0.05)	5.87 (0.01)	-3.81 (0.02)
Klucel	5.51 (0.01)	-4.86 (0.01)	5.85 (0.03)	-4.49 (0.03)	5.86 (0.01)	-3.60 (0.01)
Kollidon	5.52 (0.01)	-4.83 (0.01)	5.84 (0.04)	-4.50 (0.04)	5.85 (0.01)	-3.61 (0.01)
P-S630	5.49 (0.01)	-4.74 (0.01)	5.83 (0.01)	-4.48 (0.01)	5.86 (0.01)	-3.54 (0.02)
Without	6.51 (0.02)	-4.79 (0.02)	6.45 (0.01)	-4.20 (0.01)	6.50 (0.02)	-3.51 (0.03)
Captisol	6.55 (0.01)	-4.68 (0.01)	6.47 (0.04)	-4.02 (0.02)	6.52 (0.04)	-3.50 (0.02)
Cavasol	6.54 (0.01)	-4.80 (0.01)	6.48 (0.03)	-4.00 (0.04)	6.47 (0.01)	-3.32 (0.01)
Klucel	6.51 (0.01)	-4.73 (0.01)	6.48 (0.04)	-3.94 (0.03)	6.46 (0.01)	-3.21 (0.02)
Kollidon	6.49 (0.01)	-4.70 (0.01)	6.48 (0.03)	-3.92 (0.03)	6.46 (0.01)	-3.16 (0.04)
P-S630	6.49 (0.01)	-4.68 (0.01)	6.45 (0.01)	-3.96 (0.01)	6.45 (0.01)	-3.05 (0.04)
Without					3.53 (0.02)	-4.35 (0.03)
Captisol					3.50 (0.01)	-4.24 (0.06)
Cavasol					3.52 (0.01)	-4.37 (0.03)
Klucel					3.49 (0.05)	-4.12 (0.01)
Kollidon					3.51 (0.02)	-4.16 (0.01)
P-S630					3.49 (0.02)	-4.01 (0.01)

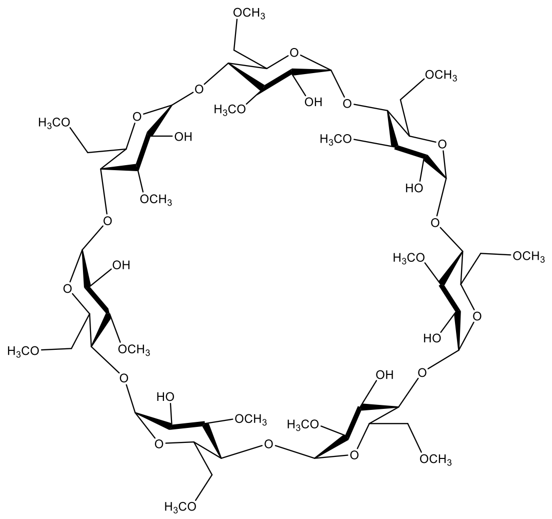

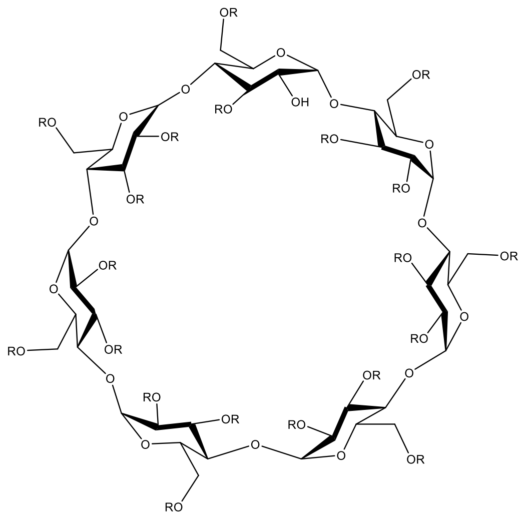

$R = (H)_{21-m}$ or $(C_4H_8SO_3^{-}Na^{+})_m$ $m = 6.0\text{-}7.1$

Cavasol

Captisol

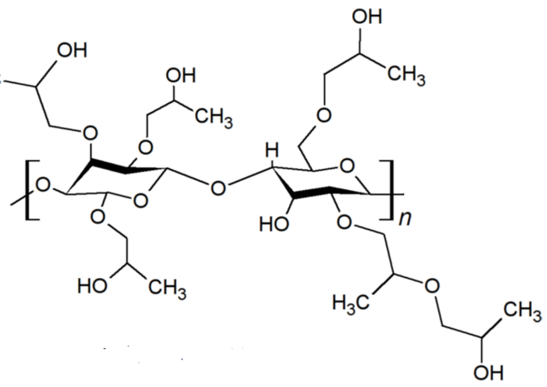

Klucel (hydroxypropylcellulose)

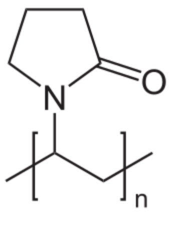

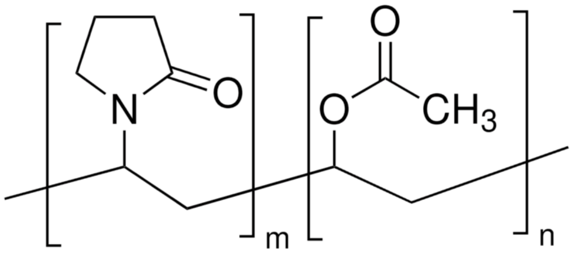

Kollidon (Polyvinylpyrrolidone)

Plasdone S630

(Polyvinylpyrrolidone + Polyvinylacetate)

Figure S1: Structures of the excipients.

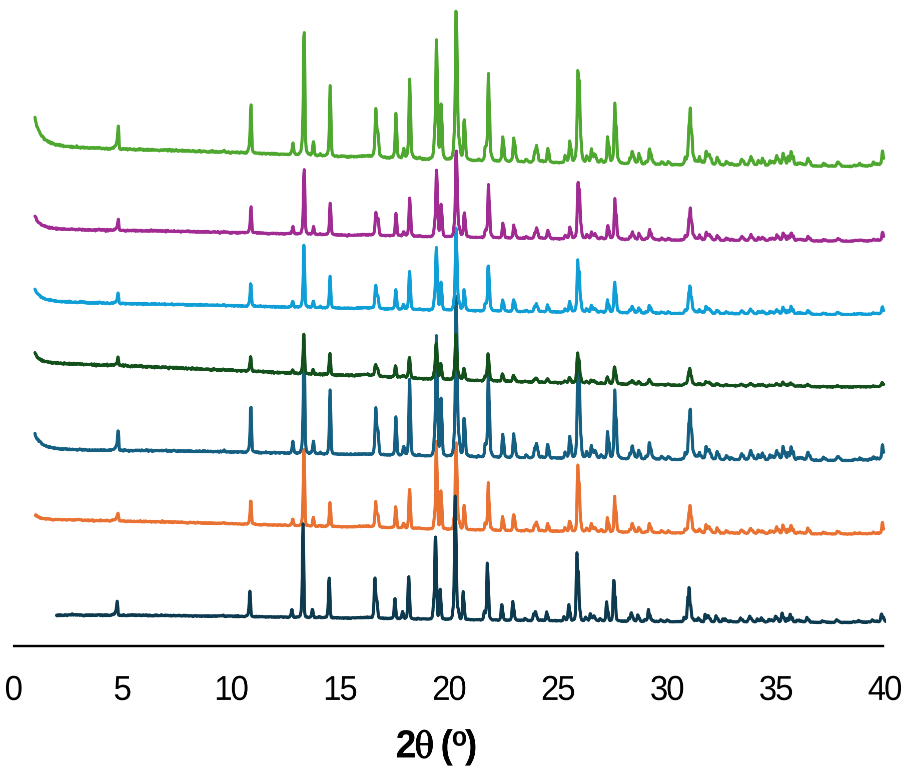

Figure S2: PXRD diffractograms for benzthiazide (Bz) raw material (●) and for the solid collected after the shake-flask process at pH 5.8: (●) free Bz; (●) Bz-CAP; (●) Bz-CAV; (●) Bz-KLU; (●) Bz-KOL; (●) Bz-S630.

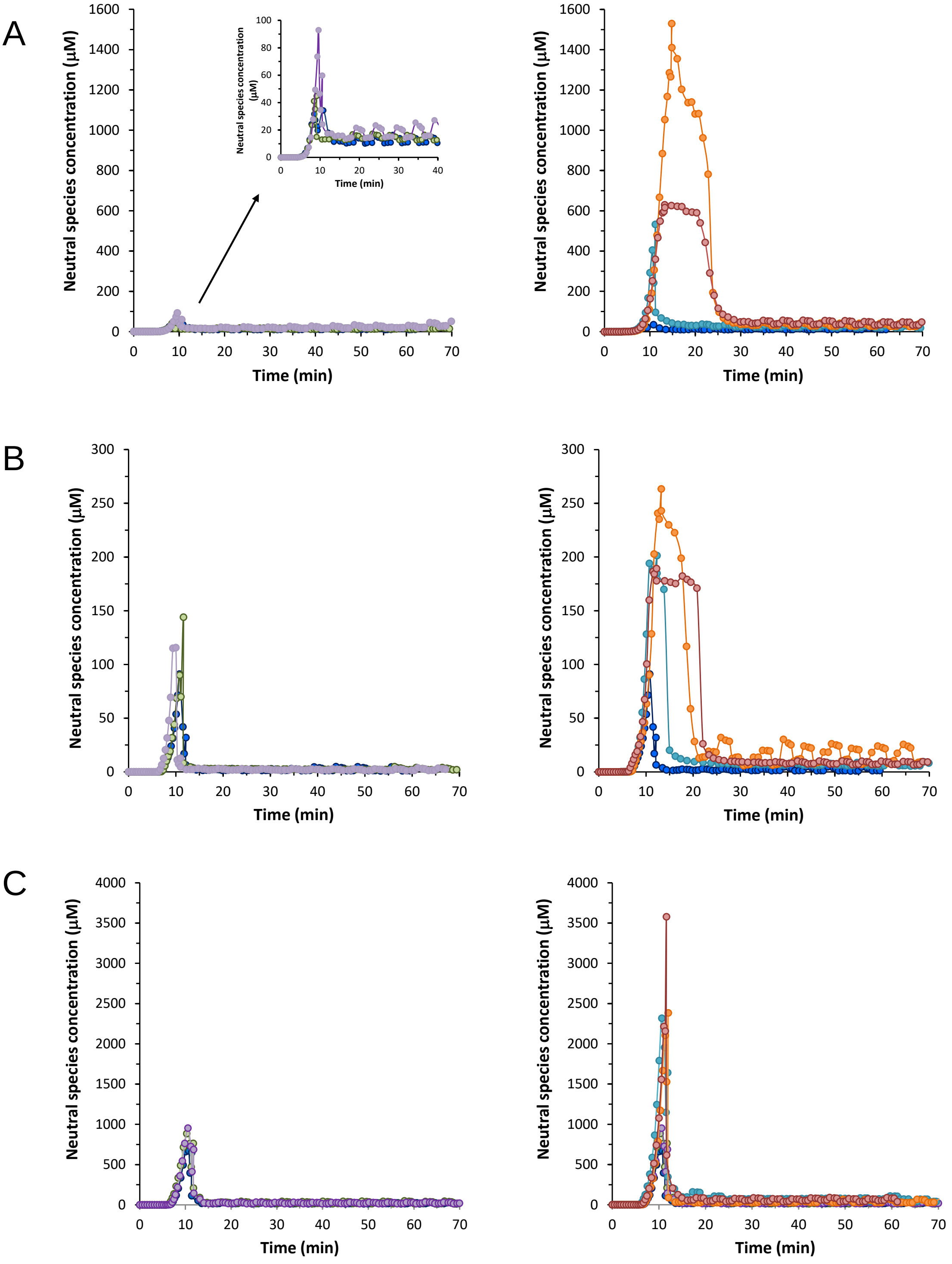

Figure S3: Supersaturation profiles for (A) benzthiazide; (B) isoxicam; (C) piroxicam. Left: (●) without excipient, (●) with captisol, (●) with cavasol. Right: (●) without excipient, (●) with klucel, (●) with kollidon, and (●) with plasdone S630.
